# iPTMnet: an integrated resource for protein post-translational modification network discovery

**DOI:** 10.1093/nar/gkx1104

**Published:** 2017-11-14

**Authors:** Hongzhan Huang, Cecilia N Arighi, Karen E Ross, Jia Ren, Gang Li, Sheng-Chih Chen, Qinghua Wang, Julie Cowart, K Vijay-Shanker, Cathy H Wu

**Affiliations:** Center for Bioinformatics and Computational Biology, University of Delaware, Newark, DE 19711, USA; Department of Computer & Information Sciences, University of Delaware, Newark, DE 19711, USA; Department of Biochemistry and Molecular & Cellular Biology, Georgetown University Medical Center, Washington, DC 20057, USA

## Abstract

Protein post-translational modifications (PTMs) play a pivotal role in numerous biological processes by modulating regulation of protein function. We have developed iPTMnet (http://proteininformationresource.org/iPTMnet) for PTM knowledge discovery, employing an integrative bioinformatics approach—combining text mining, data mining, and ontological representation to capture rich PTM information, including PTM enzyme-substrate-site relationships, PTM-specific protein-protein interactions (PPIs) and PTM conservation across species. iPTMnet encompasses data from (i) our PTM-focused text mining tools, RLIMS-P and eFIP, which extract phosphorylation information from full-scale mining of PubMed abstracts and full-length articles; (ii) a set of curated databases with experimentally observed PTMs; and iii) Protein Ontology that organizes proteins and PTM proteoforms, enabling their representation, annotation and comparison within and across species. Presently covering eight major PTM types (phosphorylation, ubiquitination, acetylation, methylation, glycosylation, S-nitrosylation, sumoylation and myristoylation), iPTMnet knowledgebase contains more than 654 500 unique PTM sites in over 62 100 proteins, along with more than 1200 PTM enzymes and over 24 300 PTM enzyme-substrate-site relations. The website supports online search, browsing, retrieval and visual analysis for scientific queries. Several examples, including functional interpretation of phosphoproteomic data, demonstrate iPTMnet as a gateway for visual exploration and systematic analysis of PTM networks and conservation, thereby enabling PTM discovery and hypothesis generation.

## INTRODUCTION

Proteins are the link between biological information in the genome and the function of cells and organisms. Protein post-translational modification, or PTM, is a major source of protein diversity. PTMs are prevalent and diverse (>620 types as cataloged in RESID (1)) and offer an additional level of regulation that allows a rapid, controlled and reversible response to environmental cues (2), for example, by dynamically altering interaction partner preferences in response to stimuli. These conditional interactions can either be mediated through single modification events, or often, through coordinated modification of multiple PTM types on multiple sites (3). To orchestrate these complex PTM events, the cell exerts tight control over the enzymes that add and remove PTMs; aberrations in PTM and PTM enzyme activity have been implicated in many diseases, including cancer (4) and neurodegeneration (5). Thus, a comprehensive picture of PTMs, as well as the multiple factors that control PTM enzyme activity are vital to understanding how cells function in health and disease.

There presently exists a number of molecular databases that focus entirely or partially on PTMs. Some cover multiple PTM types in specific taxon groups (e.g. PhosphoSitePlus (6) and PHOSIDA (7)); others focus on specific PTM types (e.g. OGlycBase (8), UniCarbKB (9), UbiProt (10), Phospho.ELM (11), PhosphoPep (12), PhosPhAt (13) and P^3^DB (14)). Some resources, such as UniProtKB (15), NeXtProt (16) and PomBase (17) are not PTM centric but have expertly curated PTM information within their entry records. Another resource, PTMcode2 (18), uses evolutionary data, structural data, and curated information from the literature to predict PTM interactions or cross-talk. dbPTM (19) integrates data from many of these resources with a special emphasis on structural aspects of the modified protein region, predicted PTM sites, and PTM sites associated with drug binding. Still, there is a need for an integrated resource that emphasizes PTM protein relationships with upstream modifying enzymes and downstream interacting partners, proteoforms(distinct molecular forms in which the protein product of a single gene can be found, including changes due to genetic variations, alternatively spliced RNA transcripts and PTMs) (20), and PTM conservation across species. Moreover, an integrated resource can play an important role in disseminating valuable PTM information in resources that are no longer being actively updated in the context of more recent information.

We have developed iPTMnet, an integrated system for protein PTM network discovery with several unique features: (i) rich up-to-date integrated PTM information from the scientific literature and knowledgebases; (ii) representation of PTM proteins, PTM enzymes and their relations at the proteoform level; (iii) score for the quality of the underlying data; (iv) network visualization of PTM enzyme-substrate-site and PPI relations and (v) sequence alignment visualization of singly-modified, multiply-modified and/or overlapping PTM forms/sites within and across species. iPTMnet connects PTM proteoforms with their modifying enzymes and multiple coordinated PTMs across taxa, thereby unifying fragmented PTM information into a biologically meaningful context for visual and systematic PTM knowledge discovery.

## MATERIALS AND METHODS

**Figure 1.**
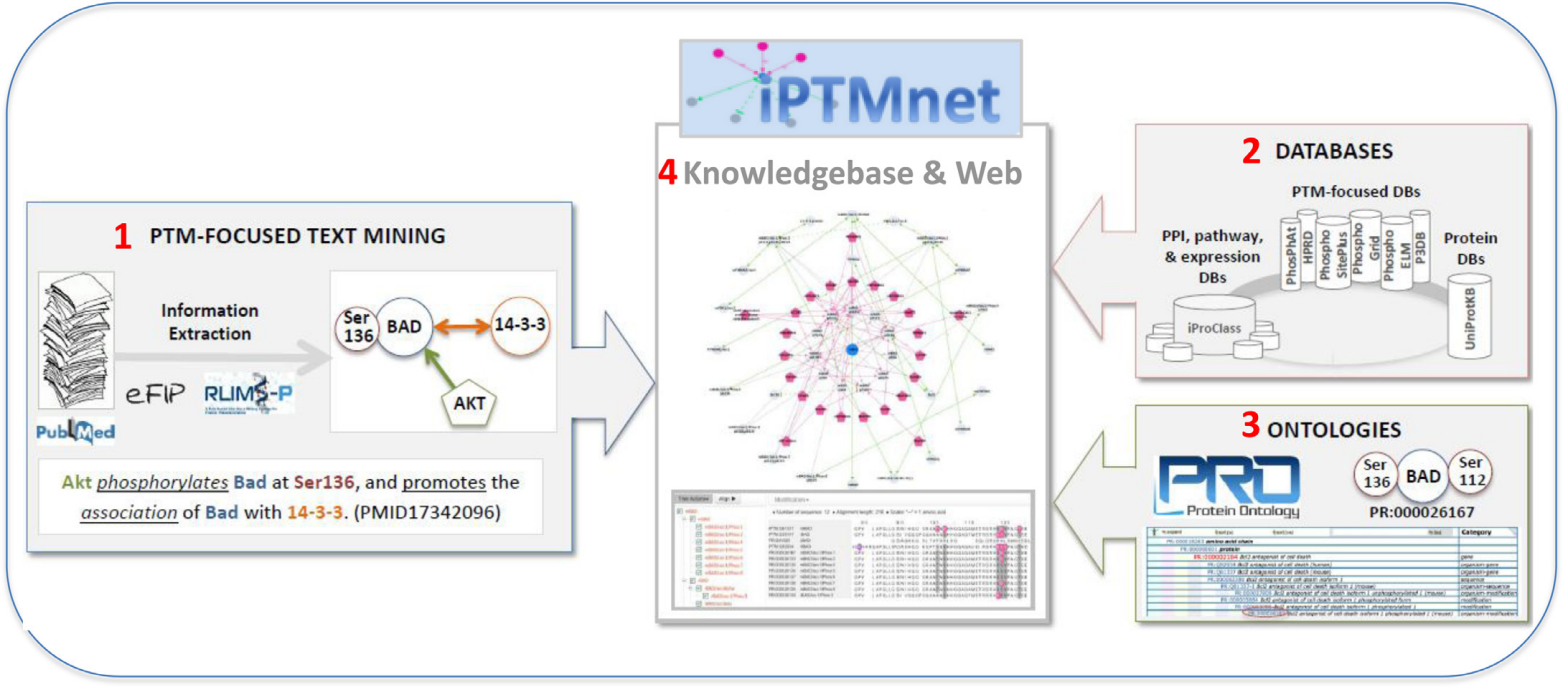
iPTMnet system components. (1) A text mining system for PTM knowledge extraction from scientific literature; (2) PTM information from high-quality manually curated databases; (3) ontologies for knowledge representation and (4) iPTMnet knowledgebase with integrated information and web portal linking the system components to support interactive scientific queries and data visualization of PTMs.

iPTMnet employs an integrative bioinformatics framework (Figure 1) to connect information from disparate bioinformatics databases, text mining results and ontologies into PTM networks for exploration and discovery. The main system components are: (i) a text mining system (see 2.1); (ii) PTM information from expert curated databases (see 2.2); (iii) Protein Ontology for proteoform representation within and across species (see 2.3) and (iv) iPTMnet knowledgebase and web portal (see 2.6). Overall, iPTMnet provides information on eight important types of PTM—phosphorylation, ubiquitination, acetylation, methylation, glycosylation, S-nitrosylation, sumoylation and myristoylation—in human and several model organisms.

### Full-Scale text mining of PTM information from literature

We have established an automated workflow for full-scale processing of all PubMed abstracts and full length articles of the PubMed Central Open Access set (PMCOA) with RLIMS-P and eFIP, which have been previously evaluated for performance and usability in community challenges (21–24). The text mining results are stored in a local database and updated on a monthly basis. The information stored includes both entities (e.g. substrate, site and interactant) and relations (e.g., kinase-substrate-site and PPI) along with the corresponding evidence in text (e.g. sentences, section IDs and PubMed IDs (PMIDs)). The annotated text is tagged with key terms for evidence attribution. In the case of the full text, we only use information extracted from the results section to increase the likelihood that the information is supported by experimental evidence in the article. To integrate the text mining results into iPTMnet, we use PubTator (25) which retrieves NCBI gene IDs linked to the protein/gene mentions.

### PTM information from curated databases

iPTMnet integrates PTM information from several expert curated resources covering both low- and high-throughput data from a range of organisms: (i) PhosphoSitePlus (PSP): expert curated PTM information including phosphorylation, ubiquitination, acetylation and methylation mainly for human, rat and mouse proteins (6); (ii) Phospho.ELM: expert curated database for phosphorylation sites in animal proteins (11); (iii) PhosPhAt: protein phosphorylation sites identified by mass spectrometry in *Arabidopsis thaliana*; (iv) PhosphoGrid: experimentally verified in vivo protein phosphorylation sites in *Saccharomyces cerevisiae* (26); (v) PomBase: a comprehensive database for the fission yeast *Schizosaccharomyces pombe*, providing structural and functional annotation, literature curation and access to large-scale data sets (17); (vi) UniProtKB: comprehensive protein database in which the reviewed section contains expert annotated information from the literature, including PTM sequence features (27); (vii) P3DB: protein phosphorylation data from multiple plants derived from large-scale experiments and the literature (14); (viii) neXtProt: a human protein knowledgebase (16) with curated proteomics and genetic variation data, particularly on kinases; ix) HPRD: PTMs and enzyme-substrate relationships for human proteins (28); (x) Signor: a database of causal relationships between biological entities, including PTM-enzyme substrate relations (29) and (xi) dbSNO: a database that integrates the experimentally verified cysteine S-nitrosylation sites from multiple species (30). Some of these databases are no longer actively curating new papers. By including them in iPTMnet, we can preserve and disseminate their valuable contribution. We maintain the integrity of this data through site/sequence validity checks and monitoring for retracted articles so that we can correct or remove information as necessary.

### Organization of proteins and PTM proteoforms using PRO

PRO constitutes a crucial part of the iPTMnet dataset as it places the PTM site information in the context of proteoforms, i.e. it shows the combination of PTMs that have been observed in a given protein. This feature of representing experimentally validated combinations of PTMs is unique. PRO uses a hierarchical representation (family→gene→sequence→modification) that depicts the relationships of a protein to its parent class and child isoforms and proteoforms. The PRO hierarchy also connects the specific proteoform of a protein across taxa when a similar modification of the conserved protein is experimentally observed in multiple organisms. Moreover, PRO provides abundant expert-curated PTM enzyme-substrate and PTM-dependent PPI relations.

All PRO data are stored in a separate database, only part of which is consumed by iPTMnet. Descriptive information for each PRO entry such as name, definition and label are pulled out directly from the PRO database. PTM, PPI, and PRO hierarchical relationships are extracted and reformatted before being imported into iPTMnet, as follows: (i) For organism-specific PTM proteoforms (PRO category: organism-modification), we extract the evidence, reference UniProtKB AC and PTM sites along with their corresponding PTM types (represented by a PSI-MOD (31) or UniCarbKB (9) identifier) from the definition of the term. PTM enzyme information is extracted from the PRO comment line; (ii) Proteoform-dependent PPI information is extracted from the PRO annotation file. Annotations with evidence_code ‘IPI’ (Inferred from physical interaction) or under the branch of ‘protein binding’ are extracted with the documented interaction partner; and iii) two types of hierarchical relations are extracted: is_a (parent-child relationship) and intersection_of (usually used to connect a proteoform with the organism it is found in).

### ID mapping for data integration

Protein/gene entries are represented in the underlying iPTMnet data sources using various names and database identifiers such as UniProtKB ACs, NCBI gene IDs, NCBI RefSeq IDs and gene symbols. We use the UniProt Protein ID and gene name mapping tools (32) to map all proteins from the source data to UniProtKB ACs. In the case of one-to-many mapping, UniProtKB/SwissProt entry is selected over TrEMBL entries to minimize redundancy. When PTM information is available from the source database at the protein isoform level, we map to the corresponding UniProtKB isoform identifier (e.g. Q15796–2 for human SMAD2, isoform 2).

### Confidence scoring of the PTM information

The quality of each piece of PTM information (e.g. a specific PTM site or PTM kinase-site relation) depends on the underlying source databases and the experimental evidence from the literature. For the latter, we consider publications as “large-scale" (LSP) if they provide information for more than 10 proteins as these papers are more likely to be reporting high throughput studies.

The confidence score (S) for each PTM information is calculated as:

\begin{equation*}{\rm{S}} = {\rm{Sq}} + {\rm{Sn}} + {\rm{Sp}},\end{equation*}

where Sq weights the quality of the underlying resource, Sn gives weight to multiple sources, and Sp gives weight to the number of publications, i.e.

\begin{equation*} \rm{Sq}=\left\{\begin{array}{l@{\quad}l} 2,& \rm{data\, from\, curated\, resources\, which\, curation\,}\\ & \rm{ policy\,ensure\, correct\, species\, assignment,} \\ 1,& \rm{data\, from\, other\, curated\, resources,} \\ 0,& \rm{data\, from\, automatic\, text\, mining;} \end{array}\right. \end{equation*}

\begin{equation*} \rm{Sn} =\left\{\begin{array}{l@{\quad}l} 1,& \rm{data\, from\, multiple\, resources,}\\ 0,& \rm{data\, from\, single\, resource;} \end{array}\right. \end{equation*}

\begin{equation*} \rm{Sp}=\left\{\begin{array}{l@{\quad}l} \,\,\,\,\,1,& \rm{data\, supported\, by\, multiple\, papers\, and\, at\,}\\& \rm{ least\,one\, is\, not\, considered\, a\, LSP,}\\ \,\,\,\,\,0,& \rm{data\, supported\, by\, one\, non-LSP\, paper,}\\ -1,& \rm{only\, LSP\, or\, no\, literature\, evidence.} \end{array}\right. \end{equation*}

The score ranges from 0 to 4, with 4 the highest confidence data. (Note we do not integrate the results from automatic text mining that are only supported by LSP.) In the iPTMnet website, the score is represented by the number of gold stars displayed next to a result.

### iPTMnet knowledgebase and web portal

We integrate text mining results, ontology and PTM database information into the iPTMnet knowledgebase. Implemented using Oracle (12c release 1), the database is designed as a dimensional model to support data retrieval and visualization. We perform integrity checks to ensure the quality of both the text mining results and database information. We verify kinase information by checking that its corresponding UniProtKB entry is annotated with the keyword ‘kinase.’ This approach is also used for other PTM enzyme types. We confirm that the assigned PTM types conform to their known residue types. PTM sites are further checked to ensure that the PTM residue is found at the expected position in the UniProtKB protein sequence of the substrate.

The front-end website is built using Django (www.djangoproject.com), a high-level Python Web Framework, which links the underlying system components to support interactive scientific queries and visualization of PTM data. The website provides two types of visualization: the Cytoscape (33) network view, implemented with the Cytoscape.js (version 2.4.2) graph theory library, and the multiple sequence alignment view. Multiple sequence alignments are performed using MUSCLE (34).

## RESULTS

### iPTMnet knowledgebase content and web dissemination

The current release of iPTMnet knowledgebase (Release 4.1, August 2017) consists of more than 654 500 unique PTM sites in 62 100 modified proteins, along with 1200 PTM enzymes (see statistics in http://research.bioinformatics.udel.edu/iptmnet/stat). There are about 12 700 distinct enzyme-substrate pairs, 24 300 distinct enzyme-substrate-site combinations, 1470 PTM-dependent PPIs, and 30 500 publications that describe one or more PTM and/or PPI relations. The top organisms represented are human, mouse, rat and *Arabidopsis*, and the top PTM types are phosphorylation, ubiquitination and acetylation. In terms of the data quality, the distribution of the confidence scores is 1.8%, 2%, 14.9%, 73.7% and 7.6% for scores from 4 (highest confidence) to 0 (lowest confidence), respectively.

The automatic text mining brings valuable data to iPTMnet as it can systematically process recent publications keeping iPTMnet up-to-date. RLIMS-P constitutes an evidence source for low-throughput experiments. For example, for TIF5A in maize (http://research.bioinformatics.udel.edu/iptmnet/entry/P80639/#asSub), the phosphorylation information for S2 and S4 from P3DB source is from a large-scale paper ‘Large-scale analysis of phosphorylated proteins in maize leaf’ (35), while RLIMS-P provides additional support for Ser2 phosphorylation from a low-throughput experiment from the article ‘Phosphorylation of maize eukaryotic translation initiation factor on Ser2 by catalytic subunit CK2’ (36). Overall, there are ∼6700 sites in iPTMnet supported by multiple sources, where RLIMS-P provides the only low-throughput evidence. Finally, RLIMS-P also provides phosphorylation data for proteins not yet curated by other sources (e.g. phosphorylation of S281 in Rtn4r, http://research.bioinformatics.udel.edu/iptmnet/entry/Q99M75/). Although RLIMS-P has been thoroughly evaluated previously, we evaluated its performance in the context of iPTMnet. We randomly selected 200 iPTMnet phosphorylation sites with RLIMS-P as an evidence source, and manually checked if the substrates and sites were correctly extracted. In this set, there were 19 results with errors (wrong substrates or sites), representing <10%, which is an error rate expected from automatic text mining. Note that the set where RLIMS-P is the unique source of evidence is more enriched for false positive results (error rate = 19% in a test of 100 randomly selected results); this set is a priority to be reviewed and corrected by iPTMnet curators. In our confidence score system, we assign results from RLIMS-P only zero stars (see section 2.5) to indicate that they are not manually curated and provide a direct link to the text evidence for easy checking.

We have developed a web portal (http://www.proteininformationresource.org/iPTMnet/) on the Protein Information Resource (PIR) website, allowing users to freely access the iPTMnet data. The iPTMnet website has received more than 6 million hits from >16 000 unique IPs in the first half of 2017.

### iPTMnet website

The iPTMnet website provides functionalities to address a variety of scientific questions. We describe below some of these functionalities along with examples. More examples can be found in (37) and in the iPTMnet on-line tutorial (http://research.bioinformatics.udel.edu/iptmnet/static/iptmnet/files/iPTMnet_Help.pdf).

#### Search and Browse Functionality

**Figure 2.**
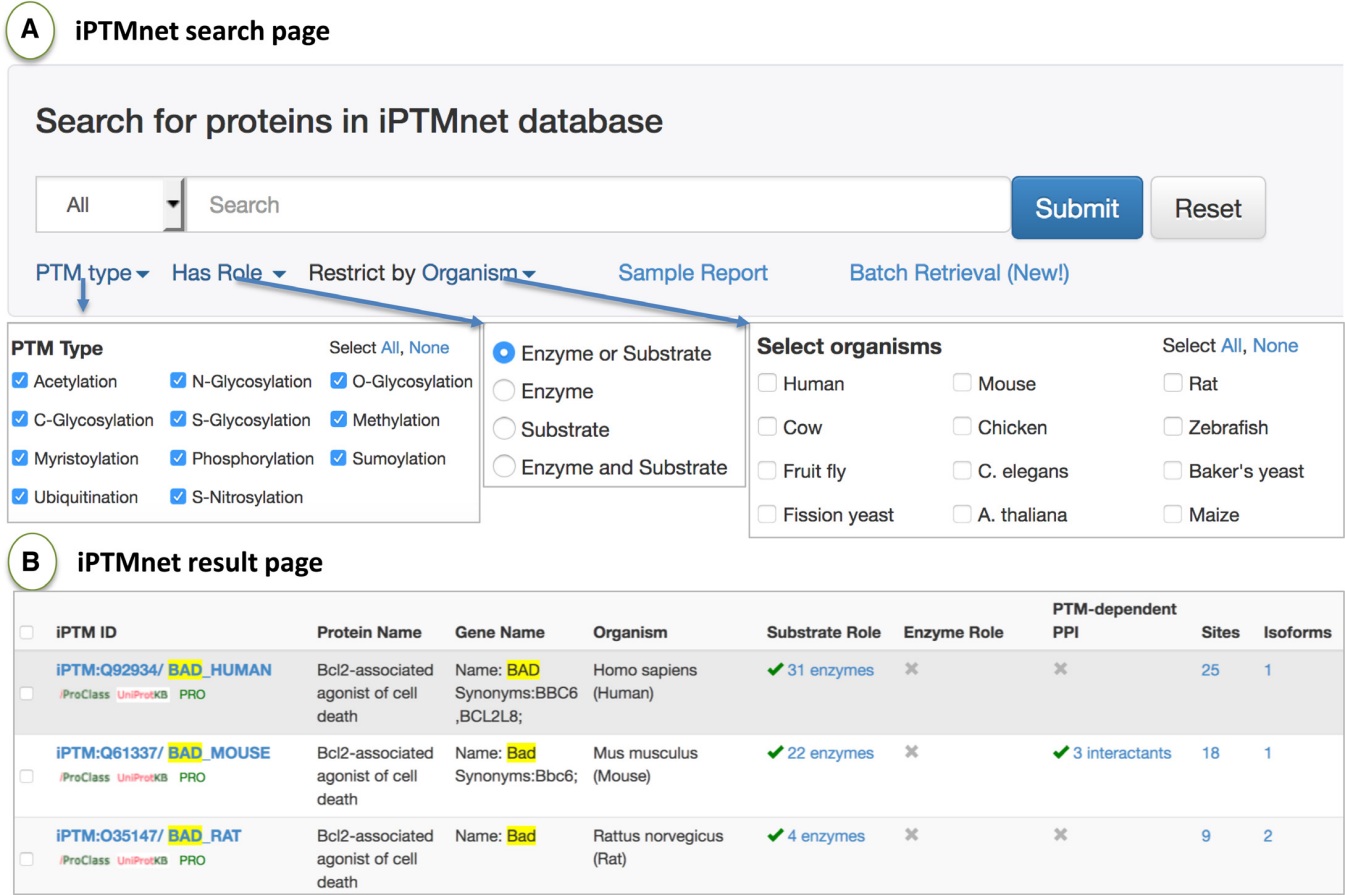
iPTMnet protein search interface. (**A**) The protein search interface allows users to search for proteins by name, accession numbers or PMID. Options to restrict search based on PTM type, role and organism are available. (**B**) The search results page displays matching results with the search terms highlighted in yellow. Results shown are for searching ‘BAD’ protein with default settings.

iPTMnet can be queried using UniProtKB AC/ID, protein or gene name, PRO ID, and PMID (Figure 2A). The search can be restricted by PTM type, enzyme or substrate role, and/or organism. The search results page returns candidate entries matching the query (matched text/ID is highlighted, Figure 2B), ordered by the overall count of all PTM data in descending order. The browse page allows the user to view entries of interest using several filters (e.g. taxon, PTM type, and role).

#### Batch retrieval

**Figure 3.**
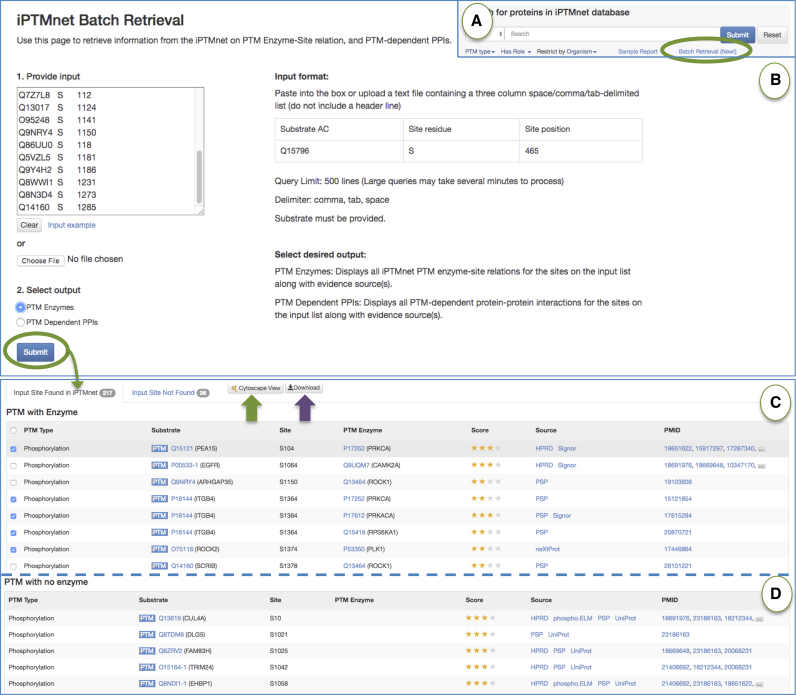
iPTMnet batch retrieval. (**A**) The batch retrieval tool can be accessed via a link under the search box (green circle). (**B**) The batch retrieval interface. Sites can be entered into the input box in a three column format—substrate AC, site residue, site position—or uploaded as a text file. The user can select to view either PTM enzymes or PTM-dependent PPIs. (**D**) Batch retrieval results showing some of the PTM enzymes for a set of 243 phosphorylation sites that are regulated by EGFR and the EGFR inhibitor, erlotinib. (**E**) Batch retrieval results showing some of the EGFR/erlotinib regulated sites that do not have PTM enzyme information.

This function allows users to obtain PTM enzyme or PTM dependent PPI information for up to 500 PTM sites at a time (Figure 3). A link to the batch retrieval page is provided on the iPTMnet home page (Figure 3A). This functionality can assist, for example, in connecting PTM sites identified in phosphoproteomic experiments to kinase signaling pathways. For example, we obtained kinase information for 243 phosphorylation sites that were down-regulated by treatment of lung cancer cells with the EGFR inhibitor, erlotinib ((4), Figure 3, Supplementary Tables S1 and S2). We retrieved 118 kinase-site pairs, with at least one kinase for 50 (20%) of the sites. These kinases belong to several pathways known to be regulated by EGFR, including PI3K/AKT/mTOR, PLC-gamma/DAG/PKC and RAS/RAF/MEK/ERK.

#### Entry report page

**Figure 4.**
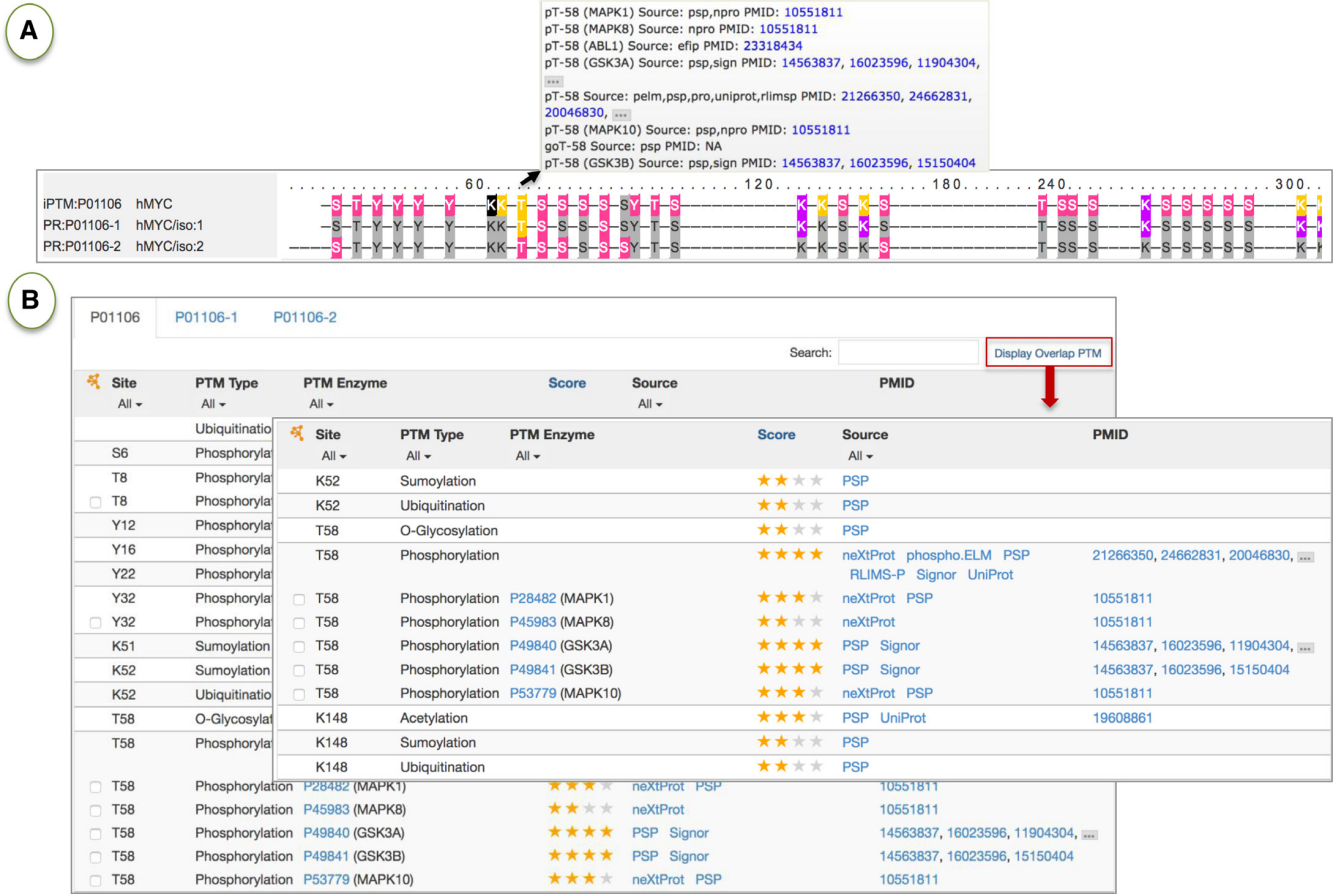
Overlapping PTMs in Myc. Selected sections of the iPTM report for MYC (http://research.bioinformatics.udel.edu/iptmnet/entry/P01106/) are shown. (**A**) Sequence viewer for human MYC sequences including isoforms with color-coded PTMs. Overlapping PTMs are highlighted in yellow. Mousing over any of these sites in the sequence shows the type modification and its source (box under arrow). (**B**) The substrate table provides further details of the experimental evidence for the modifications, including site, type of modification, PTM enzymes (if known), confidence score and sources. Results can be filtered to display only overlapping PTMs.

Clicking on an entry in the search or browse results displays the Entry Report Page for the protein. All of the data tables in the entry page are searchable and filterable. The full report (e.g.http://research.bioinformatics.udel.edu/iptmnet/entry/P31749) includes the following sections: (i) protein information from UniProt and PRO; (ii) sequence viewer with all modification sites color-coded and with link to alignment of orthologous proteoforms; (ii) substrate table indicating all PTM sites, PTM enzymes, sources and confidence score; an optional expanded view displays more detailed information about the PTM enzymes for each site and lower-confidence (zero star) results; (iii) PTM enzyme table with substrate list when applicable; (iv) proteoform table with proteoform information from PRO; (v) PTM-dependent PPI table with phosphorylated sites that affect PPI (automatically extracted using text mining tools) and (vi) proteoform-PPI table with manually curated PPIs involving PTM. Because of their potential importance in PTM cross-talk, iPTMnet highlights sites that are targets of multiple PTMs: the sequence viewer displays residues that have multiple modifications with a distinct color (yellow, Figure 4A) and the substrate table can be filtered to show only these sites (Figure 4B). For example, T58 of MYC (http://research.bioinformatics.udel.edu/iptmnet/entry/P01106/) undergoes mutually exclusive O-glycosylation and phosphorylation; these modifications are known to lead to different functional outcomes (38).

#### Interactive sequence alignment viewer

**Figure 5.**
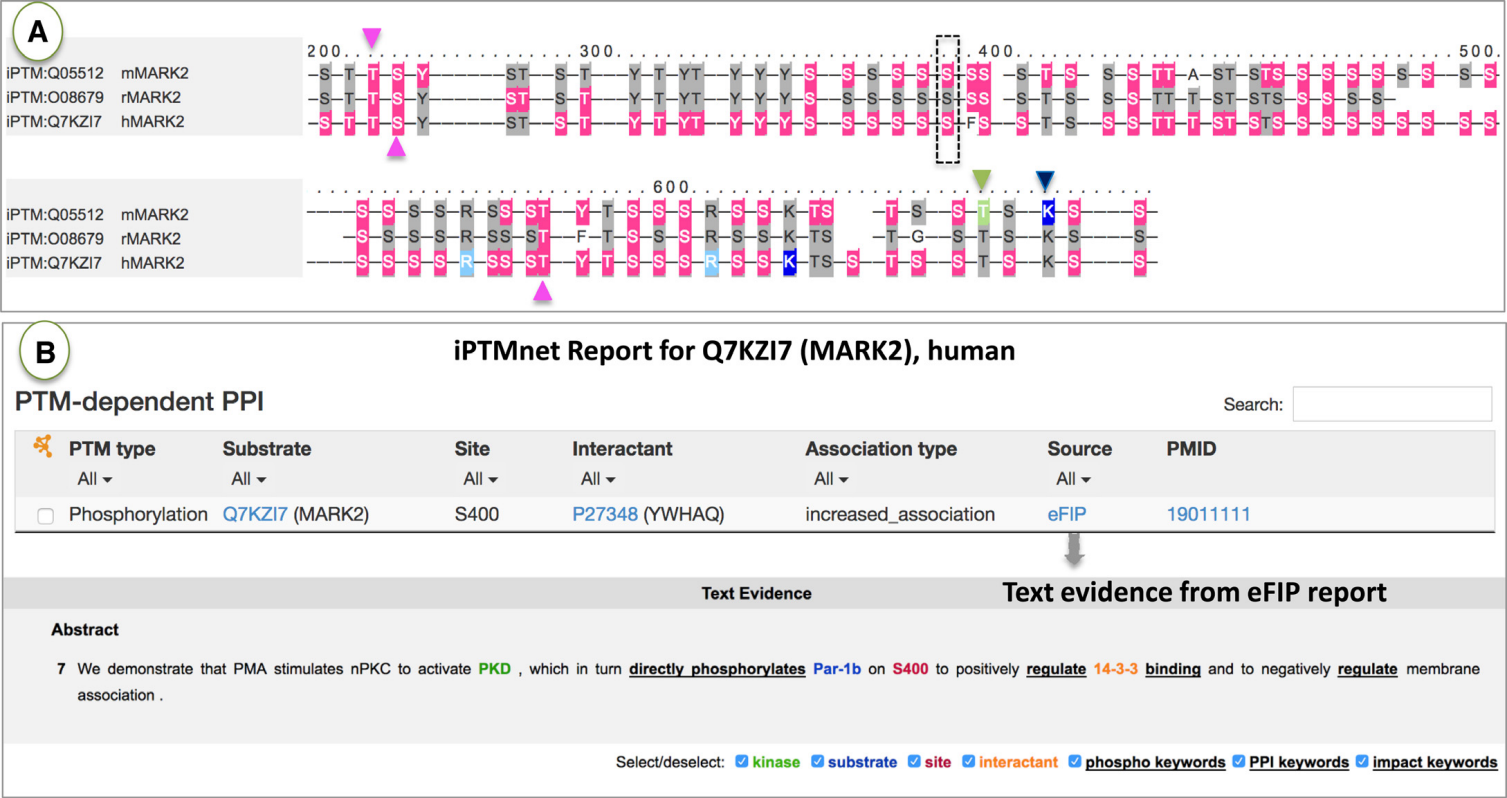
Conservation of PTM sites across species. (**A**) the interactive sequence viewer for MARK2 shows mouse, rat and human MARK2 revealing conservations of PTM sites. Sites important for activation and inhibition are indicated with magenta arrows pointing downward and upward, respectively. S400, important for phospho-dependent PPI is indicated in box. (**B**) Information on PTM-dependent PPI for human MARK2, with Text evidence of the interaction from eFIP showing the importance of phosphorylation of conserved S400.

The viewer, accessible via the entry report, allows comparison of PTMs and proteoforms across species. Very often the experimental information for PTMs in a given protein is spread over a set of related organisms, and comparing information across species can provide a more complete view of PTMs, proteoforms, and their properties. For example, the sequence alignment view of MARK2, which is a Ser/Thr protein kinase involved in cell polarity, indicates that information for PTMs exists in human, mouse and rat (Figure 5A). By inspecting the alignment, one can see that there is experimental evidence for phosphorylation of T208, an activating site (39) (magenta arrowhead pointing downward), and T596 and S212, two inhibitory sites (40,41) (magenta arrowheads pointing upwards), in all three mammalian species. The O-glycosylation (green arrowhead) and ubiquitination (blue arrowhead) sites in mouse are conserved in the orthologous sequences. More interestingly, as shown in the phospho-dependent PPI table of the human MARK2 (Figure 5B), phosphorylation of S400 has a positive impact on the association with 14-3-3 proteins. By going to the source, eFIP, we can review the text evidence for the interaction where we find that 14-3-3 negatively regulates membrane association. The mouse residue is conserved (Figure 5A, black box) and also has been shown to be phosphorylated, suggesting that it could be interesting to experimentally test whether the PTM-regulated association with 14-3-3 proteins also occurs in mouse.

#### Cytoscape network view

**Figure 6.**
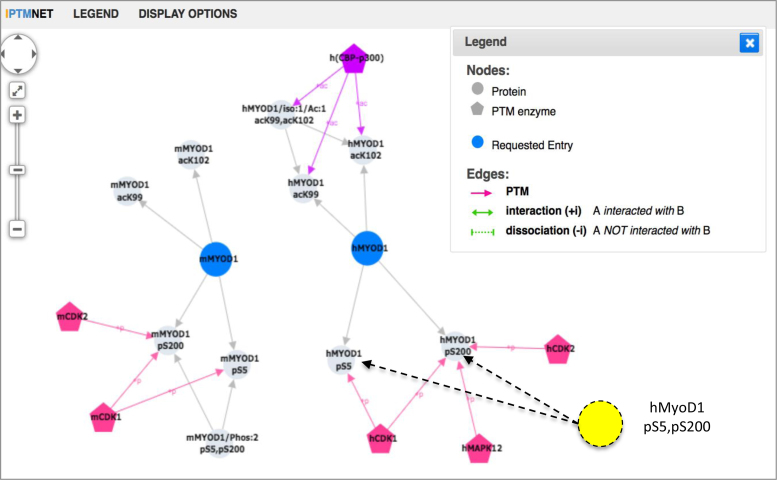
iPTMnet Network View for human and mouse MYOD1. The Cytoscape-based network view shows MYOD1 protein/proteoforms-PTM site (grey edges) and PTM enzyme-PTM site relationships (pink edges). The yellow node is a human proteoform that can be inferred based on the mouse proteoform, given that both sites and kinase involved are conserved (node added manually). To facilitate view, many other PTM nodes and edges have been hidden.

This view displays PTM sites and proteoforms as nodes, and PTM-enzyme-site, proteoform-site and PPI relationships as edges. This visualization is useful for identifying sites targeted by multiple PTM enzymes, which could be important for pathway crosstalk and PTM-dependent PPIs. Users can view the entire network for a protein or build a custom network by checking the boxes next to relations of interest in the entry and batch retrieval pages. The Cytoscape network view can provide insight into the conservation and variety of site modifications and proteoforms. For example, MYOD1, a transcriptional activator that promotes transcription of muscle-specific target genes and plays a role in muscle differentiation, has three PTM proteoforms in mouse (Figure 6). One of them, mMYOD1pS5, pS200 (PR:000029162), is phosphorylated at S5 and S200 by CDK1, decreasing MYOD1 stability (42). This doubly phosphorylated proteoform has not been described in humans. However, the iPTMnet network view shows that both S5 and S200 have been shown to be phosphorylated in human by CDK1; therefore, we can infer the existence of hMYOD1pS5, pS200, the down-regulated proteoform of MYOD1, in human (shown as a yellow node with dashed arrows in Figure 6).

#### PMID report page

Searching for a PMID opens the corresponding PMID Report Page. This page displays the title and abstract of the article followed by tables that display all of the PTM and PPI information in the iPTMnet that is derived from that article. This page serves as a point for Europe PubMed Central to link to the iPTMnet data via its external link tab (e.g., http://europepmc.org/abstract/MED/19011111?fromSearch=singleResult).

## DISCUSSION

In this work, we have described iPTMnet, a resource for PTM knowledge discovery that integrates information from the scientific literature, PTM databases, and biological ontologies, and illustrated its use through several scientific scenarios. iPTMnet has a number of key features that distinguish it from other PTM resources. These include: (i) an emphasis on experimentally validated PTM enzyme-substrate-site relations; (ii) representation of PTM proteoforms modified on combinations of sites, which can provide insight into PTM competition; (iii) information on the impact of PTM on protein function, specifically the effects of phosphorylation on PPI; and iv) alignment of orthologous proteoforms, enabling comparison of PTM proteoforms across species and prediction of PTM sites and proteoforms for proteins where experimental evidence is lacking.

Currently, iPTMnet presents text mining results for substrates and phosphorylation sites from all PubMed abstracts and the results sections of the PMCOA collection. One of the most challenging text mining tasks is gene/protein name normalization (linking protein names with database identifiers). In the current version of iPTMnet, we only integrate text mining results that have been normalized with high confidence; consequently, only a fraction of the automated results are displayed. We are improving the interface to allow users to seamlessly view the complete text mining results for a protein of interest and to integrate these results into the network view.

Other future work involves expanding the search capabilities to enable more complex queries such as: (i) retrieval of the most relevant set of substrates and enzymes given a particular PubMed-like keyword query (e.g. Alzheimer's disease); (ii) retrieval of all entries that exhibit a specified type of PTM competition (e.g. between acetylation and ubiquitination on a single site) or iii) retrieval of all entries in which PTM results in a specified functional impact (e.g. PTM-dependent PPI). We will also provide RESTful web services for the users to access the data programmatically.

In conclusion, we have shown that iPTMnet serves as a gateway for biologists to search, browse, visualize and explore PTM networks. The integrated text mining and data mining in an ontological framework connects the knowledge about the biologically relevant modified proteins from disparate data sources and the corresponding data representation enables discovery and hypothesis generation.

## Supplementary Material

Supplementary Data
